# Toward a conceptualization of decent work in Africa: Development and cross-cultural validation of the Decent Work Triad (DWT) in Burkina Faso, Switzerland, and Togo

**DOI:** 10.1177/10384162241286227

**Published:** 2024-10-08

**Authors:** Kokou A Atitsogbe, Abdoulaye Ouedraogo, Paboussoum Pari, Jérôme Rossier

**Affiliations:** 27213 University of Lausanne, Switzerland; Institut Supérieur des Sciences de la Population (ISSP), 107750 Université Joseph Ki–Zerbo, Burkina Faso; University of Lomé, Togo; 27213 University of Lausanne, Switzerland

**Keywords:** Psychology of working theory, scale development, decent work triad, contextual values, Africa

## Abstract

Psychology of Working theorists recommend supplementing the Decent Work Scale (DWS) with subjective investigations of decent work across different cultural contexts. Building on this, the present study developed and validated the Decent Work Triad (DWT) for assessing the subjective aspects of decent work in a sub-Saharan African context. Using a Burkinabe sample for exploratory (*N* = 303) and confirmatory (*N* = 494) analyses, it consists of 8 items assessing three moral and value-based dimensions: Dignity, Corruption-Free, and Shamelessness. The scale showed good psychometric properties and invariance across country and gender in Burkina Faso (*N* = 494), Switzerland (*N* = 590), and Togo (*N* = 812). It also showed incremental validity in predicting work and life satisfaction beyond the DWS and discriminant validity along with constructs of marginalization and economic constraints, underscoring its robustness. The DWT's utility in understanding the subjective and cultural dimensions of decent work and its implications were discussed.

## Introduction

The nature of work and its impact on individuals’ lives have long been subjects of scholarly interest. Consequently, decent work has become increasingly significant in global discussions about labor and employment. As the global workforce becomes increasingly diverse and interconnected, understanding the conditions constituting decent work becomes crucial for fostering equitable and fulfilling employment opportunities across different cultural contexts. According to the International Labor Organization (ILO, 1999), decent work involves productive work opportunities that deliver a fair income, security in the workplace, social protection for families, better prospects for personal development, and social integration. It also includes freedom for individuals to express their concerns, organize and participate in decisions that affect their lives, and equality of opportunity and treatment for all women and men. This comprehensive view of decent work addresses the multifaceted nature of employment and its impact on individuals and communities.

Drawing on the Psychology of Working Perspective (Blustein, 2013; Blustein et al., 2019), the Psychology of Working Theory (PWT; Duffy et al., 2016) emerged as a robust framework aimed at understanding how individuals across diverse backgrounds can achieve decent work, even in constrained environments. Building on the ILO's conceptualization, PWT offers a framework for understanding the complex interplay between work and psychological well-being. This perspective emphasizes the negative relations between social variables (i.e., marginalization and economic constraints) and decent work and the importance of work in fulfilling fundamental human needs for survival, social connection, and self-determination. PWT extends this framework by explaining how various factors influence access to decent work, including individual characteristics, contextual influences, and broader socio-economic conditions. PWT highlights that all individuals, regardless of their socio-economic background, have a right to access decent jobs, which is essential for achieving psychological well-being and social inclusion.

Central to PWT is the Decent Work Scale (DWS; Duffy et al., 2017), a 15-item measure to assess five critical dimensions of decent work: safety, healthcare, compensation, time–rest balance, and organizational and contextual value alignment. These dimensions encompass objective and subjective work aspects contributing to overall job quality and employee well-being. DWS has been validated in several countries, including a recent validation in Togo (e.g., Atitsogbe et al., 2021; Buyukgoze-Kavas & Autin, 2019; Di Fabio & Kenny, 2019; Dodd et al., 2019; Ferreira et al., 2019; Ma et al., 2023; Masdonati et al., 2019; Ribeiro et al., 2019; Vignoli et al., 2020), providing a robust measure for assessing decent work features across different contexts. However, most of the evidence underpinning its dimensions originates from ten key elements promoted by the ILO (2013) : (1) Employment opportunities, (2) Adequate earnings and productive work, (3) Decent working time, (4) Combining work, family, and personal life, (5) Work that should be abolished, (6) Stability and security of work, (7) Equal opportunity and treatment in employment, (8) Safe work environment, (9) Social security, and (10) Social dialogue. An alternative instrument based on the ILO conceptualization of decent work is the Decent Work Questionnaire (DWQ; Ferraro et al., 2018), which consists of 31 items measuring seven dimensions: Fundamental principles and values at work, Adequate working time and workload, Fulfilling and productive work, Meaningful Retribution for the exercise of citizenship, Social protection, Opportunities, and Health and safety.

Despite DWS and DWQ's international validation, most evidence supporting their utility comes from Westernized contexts. This raises critical questions about their sole applicability in non-Western settings, where cultural, economic, and social conditions can differ significantly.

## The respective contexts of Burkina Faso, Switzerland, and Togo regarding decent work

Burkina Faso, a West African Sahelian country with 20.4 million people and 63 local languages, relies heavily on agriculture, which dominates the job market (INSD, 2020; Zeye et al., 2021). Informal employment outside agriculture is exceptionally high (85.5%), with only 5% of workers in the formal sector. The job market is precarious, especially for highly educated individuals, with a high unemployment rate (23.4%) compared to uneducated individuals (1.8%). Terrorist threats over the past decade have exacerbated the scarcity of job opportunities (Moumoula et al., 2023). In Burkina Faso, working conditions are generally poor, with 85.8% of the employed population in vulnerable jobs, often independent or family work. Social protection is virtually absent, with only 7.5% of the population covered by any scheme. Due to limited public sector employment opportunities, many young people turn to entrepreneurship, with 81% viewing it as a good career choice. However, financial precarity remains a significant issue.

Switzerland has 9 million inhabitants and spans four linguistic and cultural regions: German, French, Italian, and Romansh. Hofstede's cultural dimensions describe Switzerland as an individualistic, masculine, long-term-oriented, and indulgent culture. A strong service sector and a high level of qualification among workers characterize the Swiss labor market. There is a growing trend towards part-time work. Although Switzerland scores high in job quantity, quality, and inclusiveness, specific groups such as women, foreign workers, low-skilled individuals, and young workers encounter more difficulties in the labor market (Atitsogbe et al., 2020). Careers in Switzerland are characterized by increased employment mobility across generations. In 2019, 19.2% of workers had left their jobs in the past year, with 12.7% changing employment and 6.5% becoming unemployed or exiting the labor market (Federal Statistical Office, 2020). Job changes are more frequent among younger workers and decrease with age.

Togo, bordering Burkina Faso, has a population of about 8 million and includes up to 40 ethnic languages. The informal sector employs approximately 3.5 million people (Portail Officiel de la République Togolaise, 2021). Unemployment is notably high among university graduates (32%), and decent job opportunities are limited, especially for the youth (for example, people aged less than 24 years), who constitute 60% of the population (OECD, 2016). As reviewed by Atitsogbe et al. (2019), in 2008, 40,000 applications were submitted for 3,000 public sector positions, whereas 25,586 applications were recorded in 2019 for 1,552 positions, highlighting the fierce competition for jobs. In Togo, social networking significantly influences job acquisition, with many individuals finding employment through personal connections rather than formal job search strategies. Efforts by the government to promote decent work include compulsory health care for public sector employees (with 80% healthcare coverage for their families) and students. However, more must be done to extend these benefits to the entire population, especially in the private and informal sectors.

Despite their linguistic diversity, Burkina Faso, Switzerland, and Togo share French as a common official language. In Burkina Faso and Togo, French remains the sole official language, a legacy of French colonization, whereas in Switzerland, it is one of four official languages. Moreover, despite their ethnolinguistic diversity, these countries have long been characterized by peaceful coexistence—seen in Switzerland's tradition of consensus-seeking and diplomacy—and a strong sense of national unity, as exemplified by Togo's nation-building efforts and the cohesion forged by Captain Sankara's revolution in Burkina Faso during the 1980s. Moreover, the three countries present differences in terms of formal and informal employment. As Atitsogbe et al. (2023) reviewed, 80% African jobs are in the informal sector (ILO, 2020). For instance, in Burkina Faso, 95% of jobs are informal, while in Togo, 3.5 million people work in the informal sector out of a total population of around 8 million (INSD, 2016; Portail Officiel de la République Togolaise, 2021). Such rates are higher compared to the informal economy in countries like Switzerland. The three countries present few similarities (e.g., ethnolinguistic diversity) and relatively significant differences (e.g., employment, formal/informal economy, culture).

## Toward contextual norms and values approach to decent work

Rossier and Ouedraogo's investigation (2021) on Burkinabe workers highlighted several key points. Firstly, they showed that the context significantly influences how individuals perceive their working conditions and define decent work. They argued that most workers view decent work as encompassing dignity and social recognition, aligning with the need for social connection, as outlined in the PWT (Duffy et al., 2016). This includes social support or relationships and recognition in a specific social role and space (Pouyaud, 2016). The social recognition associated with decent and meaningful work warrants further research (Urbanaviciute et al., 2019). Jobs with low social status, such as gravediggers, garbage collectors, and prostitutes, are often not seen as decent work in contexts like Burkina Faso or Togo. Additionally, jobs perceived as immoral or incompatible with workers’ community values are considered indecent, regardless of income. Rossier and Ouedraogo (2021) concluded that decent work must adhere to regulatory conditions (labor code) and align with the moral and social values of the workers’ community. Having a paid job in good working conditions promotes social integration, satisfaction, and well-being for workers, with individual visions of well-being shaped by education and cultural, moral, and religious values in context. Kazimna et al. (2020) also concluded that decent jobs should fulfill local norms and values from the Togolese participants’ perspective. Thus, contextual norms and values in the approach to decent work need to be investigated.

## The present study

Despite the significant attention that PWT has garnered from scholars worldwide, research exploring its application and implications within the African context still needs to be completed (Rossier & Ouedraogo, 2021). This gap suggests a need for further investigation to understand how cultural, social, and economic factors unique to African countries influence the components and outcomes of PWT. Expanding research in this area could provide valuable insights into the universality and adaptability of PWT and contribute to more culturally sensitive and effective interventions aimed at improving psychological well-being across diverse working populations. In response to the above-described challenges, and based on an emic approach, this study aimed to develop and validate a measure of decent work tailored explicitly to sub-Saharan African contexts. Emic approaches involve creating measurement tools grounded in the cultural realities and experiences of the studied populations. This method ensures the measured constructs are relevant and meaningful within specific cultural contexts (Leong & Pearce, 2011; Van de Vijver, 2016). Previous studies by Rossier and Ouedraogo (2021) and Kazimna et al. (2020) have underscored the importance of understanding decent work from a sub-Saharan African perspective. These studies suggest that morality, dignity, and community orientation may be more prominent in conceptualizing decent work in African settings than in Western contexts. Building on those observations, this study employed a bottom-up methodology (e.g., Atitsogbe & Bernaud, 2022) to create a culturally relevant decent work scale from a sub-Saharan African sample (Burkina Faso). It further assessed its validity across similar (i.e., Togo) and distant cultures (i.e., Switzerland). Testing the newly developed instrument in another African sample, such as Togo, will confirm its constructs’ robustness in context. Conversely, its validity in a Western context will determine whether different cultural and economic contexts endorse this conceptualization of decent work since the moral aspect of decent work has been studied less in such contexts. Furthermore, career research on dignity, morality, or ethics in the West has primarily focused on organizational behaviors or human resource management practices (e.g., ethics deficit) alienating decent work (e.g., Alzola, 2018; Bolton, 2007) rather than the moral conceptualization of decent work like the one derived from two relevant African studies (Kazimna et al., 2020; Rossier & Ouedraogo, 2021).

## Study 1: Item development and exploratory factor analysis

### Item development

Based on earlier studies regarding the conceptualization of decent work from a sub–Saharan African perspective (Kazimna et al., 2020; Rossier & Ouedraogo, 2021), we identified five categories and generated three initial items per category in French: Alignment with local norms (e.g., “I do work that aligns with the values of my community”), Social recognition (“I do work that is valued by my community”), Corruption-Free (“I do work that is honest”), Dignity (“I do dignified work”), and Shamelessness (“I do work that I am ashamed of”). Our objective guided the limited number of initial items to develop a concise unidimensional scale. The three first authors were involved in item development; two have expertise in scale development and career research. All evaluated each item's suitability, to be rated on a 7-point Likert-type scale (1 = Not true at all to 7 = Completely true).

## Exploratory factor analysis (EFA)

### Participants and procedure

We first proceeded by randomly splitting the Burkinabe sample (*N *= 607) within the large–scale cross-cultural study conducted among working adults in Burkina Faso, Togo, and Switzerland (Alfa et al., 2023) into two relatively equal samples. Sample 1 (*N *= 303) was used as the calibration sample in this study, and Sample 2 (*N *= 304) was included in the confirmatory sample with additional data (Study 2). The principle of equality regarding sex guided the splitting of the data. The calibration sample consisted of 153 (5.5%) women and 150 men (49.5%) aged 20 to 60 (*M *= 32.28; *SD *= 7.13), working in private (83.2%), public (14.5%) or parastatal companies (2.3%). Among the calibration sample, 35 individuals (11.6%) had no education (*N *= 7) or had attended only primary school (1^st^ to 6^th^ grade), 89 (29.4%) attended junior high school (7^th^ to 10^th^ grade), 62 (20.5%) attended senior high school (11^th^ to 13^th^ grade), and 117 (38.6%) attended university (bachelor's to doctoral degree).

In Burkina Faso and Togo, data collection was carried out by four doctoral students who administered a paper-based questionnaire, assisted by hired undergraduate students skilled in data collection using a convenience sampling approach. Informed consent was obtained from all participants. For those with low or no education who couldn't complete the questionnaire independently, the investigators translated the questions into local languages and recorded their responses. In Switzerland, data were gathered through both paper and online surveys. Psychology undergraduate students, as part of a research methodology course integral to their training, each collected data from about ten French-speaking working adults using their network. All respondents participated voluntarily after reviewing the study's objectives and providing their consent. In all countries, participation was uncompensated.

### Instrument

Participants answered the developed 15 items assessing decent work using a 7-point Likert–type scale ranging from 1 (Not true at all) to 7 (Completely true). Sample items measuring the five components were: “I do work that aligns with the values of my community” (Alignment with local norms), “I do work that is valued by my community” (Social recognition), “I do work that is free of corruption” (Corruption free), “I do dignified work (Dignity), and “I do work that I am ashamed of” (Shamelessness). Two of the 15 items were reversed (e.g., “I do work that I am ashamed of”). Higher scores on each component indicate higher levels of decent work from the contextual perspective.

## Results

We conducted EFA (principal axis factoring and Promax rotation) on the initial items, assuming that factors will be correlated, using Jasp .18.3.0 software. The Kaiser–Meyer–Olkin (KMO) measure of sampling adequacy was .90, surpassing the recommended threshold of .60. Additionally, Bartlett's test of sphericity was significant (*p *< .001), confirming that the data were appropriate for factor analysis (Worthington & Whittaker, 2006). We used parallel analysis to determine the number of factors to be retained (O’Connor, 2000). This procedure allows the computation of real data factor eigenvalues and simulated data mean eigenvalues. The number of factors to be retained depends on comparing both eigenvalues, especially the number of cases where real data factor eigenvalues are larger than the simulated ones. In our case, five factors should be retained. Then, we run EFA with the following item criteria: selecting items with factor loadings higher than .40, deletion of items with cross-loadings lower than .15, or presenting semantic redundancy (Worthington & Whittaker, 2006). Five items were removed using these criteria. Furthermore, along the EFA process, two items (“I do work that provides me with blessings” and “I do work that is valued by my community”) loaded each on one factor, and the respective two factors were removed, resulting in eight items loading on three factors. The first factor, Dignity, consisted of three items related to dignified work, work that is not degrading and aligns with contextual values. The second factor, Corruption–Free, consisted of three items related to honesty and having one's “hands clean” (free of corruption). The third factor, Shamelessness, consisted of two reversed items related to work that is frowned upon by others and that is a source of shame for its practitioners. The three factors explained 56.6% of the cumulative variance, with single factors explaining 28.5%, 16.8%, and 11.3% of the variance. The Decent Work Triad items and their standardized factor loadings are displayed in Table 1.

**Table 1.** Decent work triad items and standardized factor loadings.

	Factor 1	Factor 2	Factor 3
**Dignity**			
I do dignified work.	.**940**	–.037	–.071
I do work that is not degrading.	.**801**	–.023	.036
I do work that aligns with the values of my community.	.**748**	.015	.093
**Corruption–free**			
I do work that is free of corruption.	–.086	.**754**	–.055
I do work that is not dirty.	.022	.**636**	.135
I do work that is honest.	.362	.**521**	–.110
**Shamelessness**			
I do work that is looked down upon by others.^a^	–.032	.144	.**664**
I do work that I am ashamed of.^a^	.035	–.110	.**640**

*Note.* a Reversed items, factor items are bolded.

## Study 2: Confirmatory factor analysis and validity

Study 2 aimed to investigate the stability of the Decent Work Triad factor structure and its validity across the Burkinabe sample, a culturally close country, such as Togo, and a distant country, such as Switzerland. Therefore, measurement invariance across the three countries and gender were assessed. To provide evidence of predictive validity, we examined whether the newly developed Decent Work Triad predicted unique variance in job satisfaction and life satisfaction over and above the theoretically relevant outcomes of decent work as conceptualized within the Psychology of Working Theory (PWT; Duffy et al., 2016). Finally, discriminant validity will be assessed with the DWS (Duffy et al., 2017) and the constructs of marginalization and economic constraints. Thus, we hypothesize that:

H1: The Burkinabe confirmatory sample will support the three-factor structure of the Decent Work Triad.
H2: The Decent Work Triad will exhibit the three levels of invariance (configural, metric, and scalar) across countries (H2a) and gender (H2b).
H3: The Decent Work Triad will predict job satisfaction (H3a) and life satisfaction (H3b) over and above the DWS (incremental validity).
H4: It is worth noting that the newly developed DWT is a moral and value-centered approach to decent work, while the DWS could be considered a working condition-centered approach to decent work. However, a deeper examination of the DWS reveals that its Complementary Values subscale items (e.g., “My organization's values align with my family values”, “The values of my organization match the values within my community”), which assess the alignment of organizational values with family and community values, are conceptually close to the decent work conceptualization in the DWT (e.g., “I do work that aligns with the values of my community”). Thus, we expect that the DWT subscales of Dignity, Corruption-Free, and Shamelessness will correlate more strongly with the DWS Complementary Values than the other DWS subscale (H4a) (discriminant validity). Additionally, we expect the DWT scale and subscales to be negatively correlated with the constructs of marginalization and economic constraints (H4b) as theoretically posited within PWT.

## Participants

Study 2 sample consisted of Alfa et al. (2023) Burkinabe, Swiss, and Togolese samples, complemented by additional data. The Burkinabe sample consisted of 494 participants (confirmatory sample), 228 (46.2%) women, and 266 (53.8%) men aged 17 to 68 (*M *= 31.93; *SD *= 8.40). Among this Burkinabe sample, 65 individuals (13.2%) had no education (*N* = 12) or had attended only primary school (1^st^ to 6^th^ grade), 132 (26.7%) attended junior high school (7^th^ to 10^th^ grade), 131 (26.5%) attended senior high school (11^th^ to 13^th^ grade), 166 (33.6%) attended university (bachelor's to doctoral degree). The Swiss sample consisted of 590 individuals, 304 (51.5%) women, 258 (43.7%) men, and 28 (4.7%) individuals who did not disclose information about sex. They were aged 18 to 66 (*M *= 38.07; *SD *= 13.78). Among the Swiss sample, 51 individuals (8.6%) completed compulsory school (11^th^ grade), 288 (48.8%) completed high school or vocational training, and 223 (37.8%) attended university (bachelor's to doctoral degree). The Togolese sample consisted of 812 participants, 342 (42.1%) women, 469 (57.8) men, and one individual (0.1%) who did not disclose information about sex. Togolese participants were aged 13 to 65 (*M *= 34.04; *SD *= 8.81). Among the Togolese sample, 87 individuals (10.7%) had no education (n = 1) or had attended only primary school (1^st^ to 6^th^ grade), 181 (22.3%) attended junior high school (7^th^ to 10^th^ grade), 163 (20.1%) attended senior high school (11^th^ to 13^th^ grade), and 381 (46.9%) attended university (bachelor's to doctoral degree).

## Measures

### Decent work triad

The 8–item Decent Work Triad (DWT) was used. Items were rated using a 7-point Likert–type scale ranging from 1 (Not true at all) to 7 (Completely true). Sample items measuring the five components were: “I do work that aligns with the values of my community” (Alignment with local norms), “I do work that is valued by my community” (Social recognition), “I do work that is free of corruption” (Corruption-Free), “I do work that is not degrading” (Dignity), and “I do work that I am ashamed of” (Shamelessness). Two of the 15 items were reversed. Higher scores on each component indicate higher levels of decent work from the contextual perspective.

### Decent work scale

The Decent Work Scale (DWS; Duffy et al., 2017) comprises 15 items equally divided into five subscales, each corresponding to a component of decent work. We used Masdonati et al.'s (2019) French-translated version. All scales use a 7-point Likert-type response format, ranging from 1 (strongly disagree) to 7 (strongly agree). The five factors of decent work are (1) Safe working conditions (e.g., “At work, I feel safe from physical abuse”); (2) Access to healthcare (e.g., “My health insurance allows me to have access to good healthcare services”); (3) Adequate compensation (e.g., “My work is paid adequately”); (4) Free time and rest (e.g., “I do not have enough time to rest”); and (5) Complementary values (e.g., “The values of my organization are consistent with the values of my community”). A total decent work score represents the composite of the five subscale scores. Omega reliabilities for the DWS and its dimensions across the three countries ranged from .65 to .96.

### Job satisfaction scale

Job satisfaction was assessed using the JSS (Judge et al., 1998), which includes five items (e.g., “Each day of work seems like it will never end”) rated on a 7-point Likert-type scale ranging from 1 (strongly disagree) to 7 (strongly agree). The Omega reliabilities for this 5-item scale (French form) across the three countries ranged from .72 to .90.

### Satisfaction with life scale

Life satisfaction was measured using the Satisfaction With Life Scale (SWLS; Diener et al., 1985), previously adapted (French version) to the Togolese context by Sovet et al. (2016) and used in previous studies (Alfa et al., 2023; Atitsogbe et al., 2021). The adaptation consisted of rephrasing the first item (“In most ways, my life is close to my ideal”) that loaded weakly on the SWLS single factor into “Globally, my life is close to the ideal life I imagine”. This 5-item scale evaluates subjective well-being. Items were rated on a 7-point Likert-type scale ranging from 1 (strongly disagree) to 7 (strongly agree). The composite score of the five items provides an overall life satisfaction score. The Omega reliabilities for this 5-item scale across the three countries ranged from .84 to .90.

The suitability of the DWS, JSS, and SWLS for use in the Togolese and Burkinabe contexts has been previously established (Alfa et al., 2023; Atitsogbe et al., 2021**)**.

## Confirmatory factor analysis and descriptive statistics

The structural validity of the DWT was evaluated using confirmatory factor analysis (CFA) with maximum likelihood rotation in AMOS 29.0 (Table 2). Various model fit indices were examined, including χ² per degree of freedom (χ²/df), the comparative fit index (CFI), the Tucker–Lewis index (TLI), and the root mean square error of approximation (RMSEA). A good model fit should present χ²/df < 3, CFI and TLI ≥ .90 (Byrne, 2010), and RMSEA ≤ .08 or .05 (Hu & Bentler, 1999).

**Table 2.** Study 2 - confirmatory factor analyses across countries.

Scales	Burkina Faso					Switzerland					Togo				
	χ^2^/*dƒ*	CFI	TLI	RMSEA	* p*	χ^2^/*dƒ*	CFI	TLI	RMSEA	* p*	χ^2^/*dƒ*	CFI	TLI	RMSEA	* p*
Correlated 3–factor model	2.84	.983	.971	.031	< .001	3.94	.974	.958	.071	< .001	2.27	.990	.983	.040	< .001
Correlated 2–factor model^a^	7.46	.910	.868	.114	< .001	8.09	.931	.898	.110	< .001	1.95	.909	.866	.111	< .001
Correlated 2–factor model^b^	7.85	.905	.860	.118	< .001	9.41	.918	.879	.119	< .001	18.93	.836	.758	.149	< .001
Correlated 2–factor model^c^	13.17	.889	.836	.122	< .001	7.68	.935	.904	.106	< .001	13.17	.889	.836	.122	< .001
Single factor model	1.79	.857	.800	.141	< .001	11.63	.891	.847	.134	< .001	21.03	.807	.730	.157	< .001

*Note*. ^a^ Dignity and Corruption–free combined into one factor; ^b^ Corruption–free and Shamelessness combined. ^c^ Dignity and Shamelessness combined

Initially, CFA was run across the Burkinabe confirmatory sample. We established a three-factor model, three two-factor models (combining two of the three factors into one in each case), and a single-factor model, allowing each of the eight items to load on its specific factor and permitting factors to correlate. Model fit indices are summarized in Table 2. As can be seen, among all the tested models, the three-factor correlational model yielded good fit indices across the three countries: χ²/df = 2.84, CFI = .983, TLI = .971, and RMSEA = .031 (Burkina Faso). χ²/df = 3.94, CFI = .974, TLI = .958, and RMSEA = .071 (Switzerland), and χ²/df = 2.27, CFI = .99, TLI = .983, and RMSEA = .040 (Togo). This model presented standardized item loadings to corresponding factors ranging from .54 to .87 (*Mdn *= .75) for Burkina Faso, .63 to .89 (*Mdn *= .72) for Switzerland, and .58 to .86 (*Mdn *= .75) for Togo. H1 is confirmed as the DWT three-factor solution was supported. The Omega reliability coefficients for the DWT subscales of Dignity, Corruption-Free, and Shamelessness were .81, .76, and .64 for Burkina Faso, .83, .69, .73 for Switzerland, and .85, .75, and .65 for Togo. Omega for the overall scale were .83, .86, and .81 for the three countries, respectively (see Table 3). As observed, the 2-reversed item subscale of Shamelessness presented relatively low Omega values, probably due to its limited number of items. However, the overall scale presented strong Omega values across the three country samples. Descriptive statistics across countries and the overall sample are summarized in Table 4 and Table 3.

**Table 3.** Study 2 - intercorrelations between DWT, DWS, and validity constructs across the swiss and togolese samples.

	1.	2.	3.	3.1.	3.2.	3.3.	4.	4.1.	4.2.	4.3.	4.4.	4.5.	5.	6.	7.	8.	Ω	*M*	*SD*	*S*	*K*
1. Age	—	.04	.12	.12	.07	.08	–.03	–.03	.05	–.04	–.06	–.05	–.02	–.08	–.05	–.01	—	—	—	—	—
2. Sex	.00	—	–.01	.02	–.02	–.02	–.04	.00	–.02	–.09	–.08	.04	–.16	–.11	–.01	–.04	—	—	—	—	—
3. DWT	.15	–.11	—	.86	.76	.67	.04	.07	–.04	–.01	.04	.10	–.21	–.15	.03	.11	.81	5.60	1.08	–.78	.34
3.1. Dignity	.17	–.04	.89	—	.50	.43	.09	.07	.00	.01	.08	.14	–.13	–.10	.09	.18	.83	5.61	1.36	−1.13	.93
3.2. Corruption–free	.12	–.12	.85	.64	—	.21	.04	.10	–.01	–.03	–.01	.08	–.15	–.09	–.01	.02	.69	5.50	1.30	–.90	.54
3.3. Shamelessness	.07	–.10	.68	.47	.33	—	–.04	–.02	–.11	–.01	.03	–.01	–.22	–.18	–.03	.05	.73	5.76	1.61	−1.27	.74
4. DWS	.12	–.02	.43	.41	.37	.24	—	.62	.61	.72	.64	.64	.01	.06	.25	.22	.82	3.84	.87	.32	1.57
4.1. Conditions	.05	.05	.37	.34	.36	.18	.63	—	.17	.29	.22	.34	–.06	–.03	.21	.20	.80	4.13	1.32	–.04	.00
4.2. Healthcare	.09	–.08	.22	.24	.19	.09	.53	.30	—	.33	.12	.18	.08	.04	.06	.07	.87	3.47	1.61	–.04	–.70
4.3. Compensation	.09	.04	.23	.24	.18	.14	.65	.25	.15	—	.47	.29	.00	.05	.16	.15	.71	3.82	1.28	–.20	.13
4.4. Time and Rest	–.05	.02	.08	.05	.07	.08	.59	.16	.12	.20	—	.33	.02	.07	.20	.17	.68	3.87	1.28	–.04	.10
4.5. Values	.20	–.08	.47	.46	.39	.27	.7	.44	.24	.29	.21	—	–.04	.06	.26	.19	.81	3.92	1.27	–.05	.27
5. Marginalization	–.18	.02	–.29	–.23	–.25	–.22	–.3	–.27	–.19	–.16	–.16	–.19	—	.45	.07	.06	.90	3.93	1.02	–.29	.41
6. Economic constr.	–.07	.03	–.28	–.26	–.23	–.2	–.38	–.25	–.24	–.29	–.20	–.22	.46	—	.07	.05	.85	3.88	1.12	–.17	.51
7. Job satisfaction	.31	–.04	.54	.54	.39	.39	.46	.39	.20	.28	.10	.50	–.29	–.30	—	.53	.72	5.60	1.08	–.78	.34
8. Life satisfaction	.20	–.07	.39	.43	.28	.23	.50	.37	.32	.30	.20	.42	–.35	–.40	.56	—	.84	5.61	1.36	−1.13	.93
Ω	—	—	.86	.85	.75	.65	.84	.77	.92	.87	.83	.96	.93	.93	.90	.90	—	5.50	1.30	–.90	.54
*M*	—	—	6.06	6.05	5.99	6.18	5.06	5.82	5.81	4.37	4.39	4.89	5.47	5.01	6.06	6.05	—	5.76	1.61	−1.27	.74
*SD*	—	—	.89	1.03	1.09	1.15	.95	1.17	1.28	1.80	1.77	1.64	1.33	1.37	.89	1.03	—	—	—	—	—
*S*	—	—	−1.34	−1.46	−1.32	−1.83	–.50	−1.37	−1.35	–.13	–.08	–.63	−1.11	–.64	−1.34	−1.46	—	—	—	—	—
*K*	—	—	2.16	2.42	1.86	3.66	.20	1.89	1.84	−1.00	−1.01	–.36	.81	–.31	2.16	2.42	—	—	—	—	—

*Note.* Ω = Omega reliability coefficient; *S* = skewness; *K* = kurtosis. The correlations for Switzerland (N = 590) are below the diagonal and those for Togo (N = 607) are above the diagonal.

**Table 4.** Study 2 - intercorrelations between DWT, DWS, and validity constructs across the overall and burkinabe samples.

	1.	2.	3.	3.1.	3.2.	3.3.	4.	4.1.	4.2.	4.3.	4.4.	4.5.	5.	6.	7.	8.	Ω	*M*	*SD*	*S*	*K*
1. Age	—	.00	.02	.07	–.08	.05	–.05	–.02	–.09	–.14	.08	.05	–.11	–.05	.03	.03	—	—	—	—	—
2. Sex	.00	—	.05	.10	.02	.01	–.05	–.03	–.02	–.04	.01	–.05	.02	.10	.08	–.01	—	—	—	—	—
3. DWT	.14	–.04	—	.86	.82	.69	.30	.31	–.12	.13	.13	.33	–.06	–.17	.42	.20	.83	5.89	1.03	–.99	.59
3.1. Dignity	.14	.01	.87	—	.59	.41	.31	.27	–.08	.13	.14	.34	–.09	–.18	.40	.22	.81	5.94	1.21	−1.33	1.46
3.2. Corruption–free	.09	–.05	.81	.57	—	.29	.23	.25	–.07	.11	.00	.31	.01	–.07	.27	.22	.76	5.86	1.23	−1.20	1.13
3.3. Shamelessness	.09	–.05	.68	.44	.27	—	.16	.22	–.16	.07	.16	.12	–.05	–.16	.32	–.02	.64	5.84	1.56	−1.32	.88
4. DWS	.14	–.08	.27	.26	.23	.14	—	.61	.37	.53	.45	.61	–.17	–.23	.34	.39	.73	3.88	.85	–.11	–.03
4.1. Conditions	.09	–.04	.28	.24	.26	.15	.69	—	.02	.20	.07	.32	–.15	–.13	.36	.31	.80	4.82	1.59	–.58	–.35
4.2. Healthcare	.17	–.08	.06	.07	.07	.01	.64	.32	—	–.06	.01	–.08	.08	–.04	–.12	.20	.93	2.01	1.75	1.56	1.03
4.3. Compensation	.04	–.04	.13	.13	.10	.08	.62	.28	.19	—	.01	.23	–.11	–.16	.24	.25	.65	3.93	1.62	–.05	–.57
4.4. Time and Rest	–.01	–.04	.10	.10	.04	.10	.55	.20	.14	.24	—	.06	–.19	–.16	.00	–.19	.67	3.98	1.67	–.05	–.74
4.5. Values	.12	–.05	.31	.32	.28	.13	.64	.43	.15	.29	.22	—	–.07	–.12	.38	.37	.87	4.67	1.73	–.32	–.72
5. Marginalization	–.15	–.03	–.22	–.17	–.16	–.20	–.26	–.23	–.18	–.12	–.13	–.14	—	.37	–.05	.03	.86	5.10	1.14	–.36	–.17
6. Economic constr.	–.16	.04	–.24	–.20	–.16	–.21	–.37	–.30	–.34	–.18	–.15	–.17	.50	—	–.20	–.29	.84	3.76	1.52	–.11	–.80
7. Job satisfaction	.20	–.05	.37	.36	.27	.23	.49	.49	.23	.26	.15	.48	–.21	–.34	—	.39	.81	5.89	1.03	–.99	.59
8. Life satisfaction	.18	–.07	.29	.30	.21	.14	.51	.42	.40	.27	.14	.39	–.22	–.38	.55	—	.87	5.94	1.21	−1.33	1.46
Ω	—	—	.83	.83	.73	.69	.83	.83	.95	.75	.73	.89	.90	.90	.84	.88		5.86	1.23	−1.20	1.13
*M*	—	—	5.82	5.83	5.75	5.91	4.23	4.83	3.82	4.02	4.06	4.42	4.76	4.29	5.82	5.83		5.84	1.56	−1.32	.88
*SD*	—	—	1.03	1.24	1.24	1.48	1.05	1.53	2.14	1.57	1.56	1.58	1.37	1.42	1.03	1.24	—	—	—	—	—
*S*	—	—	–.99	−1.31	−1.10	−1.46	.18	–.43	–.03	.00	.04	–.15	–.26	–.19	–.99	−1.31	—	—	—	—	—
*K*	—	—	.76	1.57	.98	1.48	–.11	–.51	−1.36	–.46	–.53	–.53	–.50	–.46	.76	1.57	—	—	—	—	—

*Note.* Ω = Omega reliability coefficient; *S* = skewness; *K* = kurtosis. The correlations for the overall sample (N = 1896) are below the diagonal. and those for Burkina Faso (N = 494) are above the diagonal.

Intercorrelations between the DWT scales and subscales were positive and significant and ranged from .29 to .86 across the Burkinabe sample, from .33 to .89 across the Swiss sample, .21 to .86 across the Togolese sample, and from .27 to .87 across the overall sample. It should be noted that the highest correlations were observed between the DWT total score and the Dignity subscale score.

## Measurement invariance

This study also assesses measurement invariance to ensure the DWT is valid and reliable across different cultural settings. Measurement invariance refers to how much a measure assesses the same construct across various groups. Establishing measurement invariance is crucial for making meaningful comparisons between groups and ensuring that any observed differences are due to actual differences in the measured construct rather than artifacts of the measurement process. Multi-group confirmatory factor analyses (MGCFAs) were conducted to assess the configural, metric, and scalar invariance of the Decent Work Triad across the three countries and their respective gender groups (see Atitsogbe et al., 2018 for an example of this approach), using AMOS 29.0. The model fit was evaluated by inspecting changes in model fit statistics (Vandenberg & Lance, 2000), changes in CFI, which should be less than .01 (Cheung & Rensvold, 2002) or less than .002 according to Meade et al., (2008), and changes in RMSEA, which should be lower than .05 (e.g., Savickas & Porfeli, 2012).

Configural, metric, and scalar invariance were achieved between Burkina Faso and Togo, Burkina Faso and Switzerland, and between Switzerland and Togo, with ΔCFI and ΔRMSEA always being equal to or less than .01 and .05, respectively (see Table 5). CFIs and TLIs were consistently above .90, χ²/df values were slightly above 3 in most cases but lower than 3 within the comparison between Burkina Faso and Togo. Findings suggested that the scale reached perfect invariance across the three countries. Therefore, H2a regarding the DWT cross-country invariance was supported. Furthermore, the three levels of measurement invariance (configural, metric, and scalar) were achieved regarding gender in each country, supporting H2b.

**Table 5.** Measurement invariance for the decent work triad three-factor structure across countries and gender.

	χ^2^	*dƒ*	χ^2^/*dƒ*	*p*	CFI	TLI	RMSEA	Δχ^2^	Δ*d*ƒ	*p*	ΔCFI	ΔTLI	ΔRMSEA
Measurement invariance across countries													
BF and CH													
Configural Invariance	106.42	34	3.13	< .001	.978	.964	.044						
Metric Invariance	109.44	39	2.81	< .001	.979	.970	.041	3.02	5	.697	.001	.006	.003
Scalar Invariance	138.68	45	3.08	< .001	.972	.965	.044	29.24	6	< .001	.007	.005	.003
BF and TG													
Configural Invariance	78.01	34	2.30	< .001	.987	.979	.032						
Metric Invariance	92.92	39	2.38	< .001	.984	.978	.033	14.91	5	.011	.003	.001	.001
Scalar Invariance	11.20	45	2.45	< .001	.981	.976	.033	17.28	6	.008	.003	.002	.000
CH and TG													
Configural Invariance	105.55	34	3.10	< .001	.982	.971	.039						
Metric Invariance	117.93	39	3.02	< .001	.980	.972	.038	12.38	5	.030	.002	.001	.001
Scalar Invariance	222.38	45	4.94	< .001	.956	.945	.053	104.45	6	<.001	.001	.027	.015
Measurement invariance across gender														
BF													
Configural Invariance	58.03	34	1.71	.006	.982	.971	.038						
Metric Invariance	64.51	39	1.65	.006	.981	.973	.036	6.48	5	.262	.001	.002	.002
Scalar Invariance	7.30	45	1.56	.009	.982	.977	.034	5.79	6	.447	.001	.004	.002
CH													
Configural Invariance	104.78	34	3.08	< .001	.986	.978	.033						
Metric Invariance	117.47	39	3.01	< .001	.985	.978	.033	12.69	5	.026	.001	.000	.000
Scalar Invariance	137.28	45	3.05	< .001	.982	.978	.033	19.81	6	.002	.003	.000	.000
TG													
Configural Invariance	77.83	34	2.29	< .001	.979	.966	.040						
Metric Invariance	87.55	39	2.25	< .001	.977	.967	.039	9.72	5	.084	.002	.001	.001
Scalar Invariance	104.27	45	2.32	< .001	.972	.965	.040	16.72	6	.010	.005	.002	.001

*Note*. BF = Burkina Faso; CH = Switzerland; TG = Togo.

## Predictive and discriminant validity

To provide validity evidence of the DWT, we investigated incremental validity and discriminant validity across countries. According to Weiner and Greene (2007), incremental validity should address whether the scale of interest provides additional information beyond what previous variables can predict, and discriminant validity evaluates how unrelated a measure is to other measures measuring dissimilar constructs.

We performed a hierarchical regression to assess the incremental validity of the DWT in predicting job satisfaction (H3a) and life satisfaction (H3b) across the three country samples. Age was entered in the first step (as it correlated more with the study variables than sex), followed by the five DWS components in the second step. In the third step, previous steps were continued, adding the DWT components to evaluate the incremental validity of DWT over DWS. After controlling for age, DWS explained 21%, 34%, and 11% variance of job satisfaction in the Burkinabe, Swiss, and Togolese samples, respectively, and 26%, 27%, and 8% variance of life satisfaction. The additional effect of DWT in explaining job satisfaction was particularly important, especially across the Burkinabe and Swiss samples: Burkina Faso, ΔR^2 ^= 0.09, F(3,293) = 12.73, *p *< 0.001; Switzerland, ΔR^2 ^= 0.10, F(3,556) = 34.78, *p *< 0.001; Togo, ΔR^2 ^= 0.01, F(3,622) = 1.85, *p *= 0.14, partially supporting H3a. Furthermore, the additional effect of DWT in explaining life satisfaction was important, especially across the Swiss and Togolese samples: Burkina Faso, ΔR^2 ^= 0.02, *F*(3,293) = 2.01, *p *= .11; Switzerland, ΔR^2 ^= 0.05, *F*(3,556) = 12.43, *p *< .001; Togo, ΔR^2 ^= 0.01, *F*(3,622) = 7.00, *p *< .001, partially supporting H3b. However, findings showed that DWT had a significant incremental validity regarding the predictive power of DWS on both job satisfaction and life satisfaction across countries. The results are shown in Table 6.

**Table 6.** Study 2 - hierarchical regression predicting DWT and DWS from marginalization and economic constraints.

	Burkina Faso						Switzerland						Togo					
	Job satisfaction			Life satisfaction			Job satisfaction			Life satisfaction			Job satisfaction			Life satisfaction		
	β	SE	*t*	β	SE	*t*	β	SE	*t*	β	SE	*t*	β	SE	*t*	β	SE	*t*
**Model 1**																		
Age	0.03	0.01	0.60	0.03	0.01	0.50	**0**.**31^c^**	0.00	7.86	**0**.**20^c^**	.00	4.93	−0.04	0.01	−0.97	0.01	0.00	0.14
R^2^		<.01			<.01			0.10			0.04			0.00			<.01	
**Model 2**																		
Age	0.04	0.01	0.68	0.07	0.01	1.40	**0**.**22^c^**	0.00	6.33	**0**.**13^c^**	0.00	3.50	−0.03	0.00	−0.71	0.01	0.00	0.89
Conditions	0.26^c^	0.04	4.69	**0**.**15^c^**	0.05	2.88	**0**.**21^c^**	0.05	5.22	**0**.**17^c^**	0.05	4.06	**0**.**12^b^**	0.03	2.65	**0**.**13^c^**	0.04	0.00
Healthcare	**−0**.**11^a^**	0.03	−2.11	**0**.**22^c^**	0.04	4.29	0.03	0.04	0.72	**0**.**17^c^**	0.04	4.46	−0.01	0.03	−0.32	0.00	0.03	0.95
Compensation	0.13^a^	0.04	2.44	**0**.**15^c^**	0.05	2.84	**0**.**10^b^**	0.03	2.83	**0**.**13^c^**	0.03	3.41	0.01	0.04	0.11	0.02	0.05	0.68
Time and Rest	0.00	0.04	0.04	**−0**.**18^c^**	0.05	−3.51	−0.03	0.03	−0.87	**0**.**09^b^**	0.03	2.31	0.07	0.04	1.50	0.06	0.05	0.20
Values	0.25^c^	0.04	4.46	**0**.**30^c^**	0.05	5.56	**0**.**33^c^**	0.03	8.28	**0**.**21^c^**	0.03	5.05	0.19^c^	0.04	4.24	0.11^a^	0.04	0.01
R^2^		0.21			0.26			0.34			0.27			0.11			0.08	
Δ R^2^		0.23			0.27			0.25			0.24			0.12			0.09	
**Model 3**																		
Age	0.02	0.01	0.32	0.07	0.01	1.38	**0**.**20^a^**	0.00	6.21	**0**.**11^c^**	0.00	3.16	−0.04	0.00	−0.89	−0.02	0.00	−0.53
Conditions	**0**.**21^c^**	0.04	3.93	**0**.**15^c^**	0.06	2.73	**0**.**16^a^**	0.04	4.23	**0**.**14^c^**	0.05	3.52	**0**.**12^b^**	0.03	2.73	**0**.**14^c^**	0.04	3.15
Healthcare	−0.03	0.03	−0.67	**0**.**22^c^**	0.04	4.24	0.00	0.04	−0.03	**0**.**15^c^**	0.04	4.01	−0.01	0.03	−0.35	0.01	0.03	0.24
Compensation	**0**.**13^b^**	0.04	2.49	**0**.**14^a^**	0.05	2.70	**0**.**07^a^**	0.03	2.11	**0**.**11^c^**	0.03	2.90	0.01	0.04	0.10	0.02	0.05	0.31
Time and Rest	−0.03	0.04	−0.69	**−0**.**18^c^**	0.05	−3.53	−0.02	0.03	−0.45	**0**.**10^b^**	0.03	2.75	0.07	0.04	1.41	0.05	0.05	1.07
Values	**0**.**18^c^**	0.04	3.23	**0**.**25^c^**	0.05	4.40	**0**.**20^c^**	0.03	5.17	**0**.**13^c^**	0.04	3.10	**0**.**18^c^**	0.04	3.94	**0**.**09^a^**	0.04	2.03
Dignity	**0**.**24^c^**	0.06	3.72	**0**.**11^b^**	0.09	1.73	**0**.**27^c^**	0.06	5.94	**0**.**26^c^**	0.07	5.04	**0**.**11^b^**	0.04	2.34	**0**.**20^c^**	0.04	4.26
Corruption-Free	−0.05	0.06	−0.81	0.04	0.08	0.68	0.00	0.05	0.10	−0.07	0.06	−1.50	−0.05	0.03	−1.15	−0.08	0.04	−1.77
Shamelessness	**0**.**19^c^**	0.04	3.63	−0.05	0.06	−0.84	**0**.**14^c^**	0.04	3.97	0.04	0.05	0.95	−0.05	0.03	−1.06	0.00	0.03	−0.11
R^2^		0.30			0.27			0.44			0.31			0.11			0.08	
Δ R^2^		0.09			0.02			0.10			0.05			0.01			0.01	

*Note.* ^a^ *p *< .05. ^b^ *p *< .01. ^c^ *p *< .001. Significant regressions are bolded.

Regarding discriminant validity (see Table 4 and Table 3), as hypothesized, the DWT subscales of Dignity, Corruption-Free, and Shamelessness correlated more strongly with the DWS Complementary Values Subscale (BF: r = .34; CH: r = .46; TG: r = .14;) than the other DWS subscales of Working Conditions (BF: r_Dignity _= .27; r_Corruption−Free _= .25; r_Shamelessness _= .22; CH: r_Dignity _= .34; r_Corruption−Free _= .36; r_Shamelessness _= .18; TG: r_Dignity _= .07; r_Corruption−Free _= .00; r_Shamelessness _= .01), Healthcare (BF: r_Dignity _= –.12; r_Corruption−Free _= –.08; r_Shamelessness _= –.07; CH: r_Dignity _= .24; r_Corruption−Free _= .19; r_Shamelessness _= .09; TG: r_Dignity _= .00; r_Corruption−Free _= −.01; r_Shamelessness _= –.11), Compensation (BF: r_Dignity _= .13; r_Corruption−Free _= .11; r_Shamelessness _= .07; CH: r_Dignity _= .24; r_Corruption−Free _= .18; r_Shamelessness _= .14; TG: r_Dignity _= .01; r_Corruption−Free _= –.03; r_Shamelessness _= –.01), and Free and Rest (BF: r_Dignity _= .14; r_Corruption−Free _= .00; r_Shamelessness _= .16; CH: r_Dignity _= .05; r_Corruption−Free _= .07; r_Shamelessness _= .08; TG: r_Dignity _= .08; r_Corruption−Free _= –.01; r_Shamelessness _= .03). These results fully support H4a. Regarding the expected negative correlations between DWT and the constructs of marginalization and economic constraints (H4b), results generally support our assumption regarding these dissimilar constructs. As can be seen in Table 4 and Table 3, correlations between DWT total score or subscale scores ranged from –.09 to .01 (marginalization) and from –.18 to –.07 (economic constraints) across the Burkinabe sample; –.29 to –.22 (marginalization) and from –.28 to –.20 (economic constraints) across the Swiss sample; and –.22 to –.13 (marginalization) and from –.18 to –.09 (economic constraints) across the Togolese, indicating that DWT measures a construct not similar to marginalization and economic constraints.

## General discussion

The present study originally aimed to develop a psychometrically sound scale to measure decent work from a subjective and contextually circumscribed perspective stemming from Burkina Faso, with further validation in the close and distant contexts of Togo and Switzerland, respectively. The newly developed 8-item Decent Work Triad assesses three domains of decent work from a context-based perspective in Africa: Dignity, Corruption-Free, and Shamelessness. The study complements the existing measures of decent work based on the ILO conceptualization (Duffy et al., 2017; Ferraro et al., 2018). It also contributes to the existing decent work literature, which has focused primarily on ILO indicators.

The identified three-factor solution in the Burkinabe validation sample replicated well in a culturally close context (Togo) and a distant culture (Switzerland). The three levels of invariance (i.e., configural, metric, and scalar) were achieved across the three countries. Moreover, the three levels of invariance were reached in each country for gender. Such results argue the universality of the measured decent work features (i.e., Dignity, Corruption-Free, and Shamelessness). However, the expression of those dimensions would be moderated by context. Thus, although developed in an African setting, the DWT showed validity for use in Switzerland. Further studies could provide more insight into its applicability in other contexts. As scalar invariance was reached across the three countries, further studies could investigate gender mean comparisons.

Regarding the two other features of decent work within DWT, Dignity and Corruption-Free are cultural and moral values grounded in Burkina Faso's history, for example, the revolution led by Captain Thoma Sankara in the 1980s that resulted in renaming the country “Burkina Faso”. The name “Burkina Faso” originates from two predominant local languages in the country. “Burkina” is derived from the Mooré language and carries multiple meanings, including honor, dignity, respect, and honesty, values among others, observed as norms in Africa (Atitsogbe & Bernaud, 2022). The term “Faso” is from the Dioula language, translating to Fatherland or Republic. Consequently, “Burkina Faso” signifies “the country of people of integrity, honesty, courage, and respect” (Kyelem de Tembela, 2012), also designated by the French expression “Pays des hommes intègres” [Country of upright men]. This name change encapsulated a comprehensive vision for establishing a new society, one where the core values of dignity and integrity are paramount. Such values are expected not only from individuals in the society but also from the leaders. As such, dignity, integrity, and the necessary emancipation are frequently cited among motives for recent successive coups d'état in Burkina Faso and other African countries, including Niger and Mali (Korotayev et al., 2024; Ouedraogo, 2024). These values are anticipated to guide all facets of life for economic and social stability in these countries and broadly in Africa, influencing even the nature of work.

Moreover, the findings have practical implications for policymakers, employers, and labor organizations promoting decent work in collectivistic and conservative cultures. By identifying culturally specific factors influencing perceptions of decent work, interventions can be tailored to address African workers’ unique needs and priorities. This effort not only advances theoretical understanding but also has the potential to inform policies and practices that promote equitable and fulfilling employment opportunities for all workers, regardless of their cultural background. Research suggests that career interventions can enhance access to decent work, leading to positive spillover effects on establishing meaningful work and decent lives (Di Fabio & Blustein, 2016; Di Fabio et al., 2023; Kenny & Di Fabio, 2023; Duffy et al., 2016). Therefore, vocational guidance and career counseling interventions should incorporate the moral and value-based perspective outlined in the DWT, particularly in conservative contexts. These interventions should consider how clients in careers that deviate from societal values and norms (e.g., garbage collectors, sex workers) manage or navigate their careers in environments with conservative pressures.

While offering valuable insights into the moral and value-based conceptualization of decent work, the study presents several limitations. The first limitation lies in the sampling technique. Using convenience samples could not guarantee that participants in each of the three countries are representative of the local working adult population in terms of education or formal versus informal sectors. For example, although they have been included in the current study, people with no education seem to have been underestimated within the Burkinabe and Togolese samples. Both countries present higher rates of adults with no education: 34% literacy rate in Burkinabe (year 2022) and 67% in Togolese (year 2019) above 15 years old (World Bank, 2024). Further research using representative samples could increase confidence in the results. The second limitation lies in the fact that the stability of the DWT should be considered in light of the specific studied contexts. By incorporating local specificities into the new scale, the study enhances our understanding of what constitutes decent work in non-westernized cultural contexts of Sub-Saharan Africa. Interestingly, the DWT was found to be stable in the western sample of Switzerland, indicating that individuals in this geographically distant area also align with the moral or value-based perspective outlined in the DWT. However, further research is needed to assess the model's universality. Additional studies examining the DWT's validity in other Western contexts could offer deeper insights into the global applicability of this perspective.
